# Bridged-U-Net-ASPP-EVO and Deep Learning Optimization for Brain Tumor Segmentation

**DOI:** 10.3390/diagnostics13162633

**Published:** 2023-08-09

**Authors:** Rammah Yousef, Shakir Khan, Gaurav Gupta, Bader M. Albahlal, Saad Abdullah Alajlan, Aleem Ali

**Affiliations:** 1 Yogananda School of AI, Computers and Data Sciences, Shoolini University, Solan 173229, India; 2 College of Computer and Information Sciences, Imam Mohammad Ibn Saud Islamic University (IMSIU), Riyadh 11432, Saudi Arabiasaalajlan@imamu.edu.sa (S.A.A.); 3 Department of Computer Science and Engineering, University Centre for Research and Development, Chandigarh University, Mohali 140413, India

**Keywords:** Bridged U-Net, BraTS 2020–2021 dataset, spatial pyramid pooling, brain tumor segmentation

## Abstract

Brain tumor segmentation from Magnetic Resonance Images (MRI) is considered a big challenge due to the complexity of brain tumor tissues, and segmenting these tissues from the healthy tissues is an even more tedious challenge when manual segmentation is undertaken by radiologists. In this paper, we have presented an experimental approach to emphasize the impact and effectiveness of deep learning elements like optimizers and loss functions towards a deep learning optimal solution for brain tumor segmentation. We evaluated our performance results on the most popular brain tumor datasets (MICCAI BraTS 2020 and RSNA-ASNR-MICCAI BraTS 2021). Furthermore, a new Bridged U-Net-ASPP-EVO was introduced that exploits Atrous Spatial Pyramid Pooling to enhance capturing multi-scale information to help in segmenting different tumor sizes, Evolving Normalization layers, squeeze and excitation residual blocks, and the max-average pooling for down sampling. Two variants of this architecture were constructed (Bridged U-Net_ASPP_EVO v1 and Bridged U-Net_ASPP_EVO v2). The best results were achieved using these two models when compared with other state-of-the-art models; we have achieved average segmentation dice scores of 0.84, 0.85, and 0.91 from variant1, and 0.83, 0.86, and 0.92 from v2 for the Enhanced Tumor (ET), Tumor Core (TC), and Whole Tumor (WT) tumor sub-regions, respectively, in the BraTS 2021validation dataset.

## 1. Introduction

**Figure 1:**
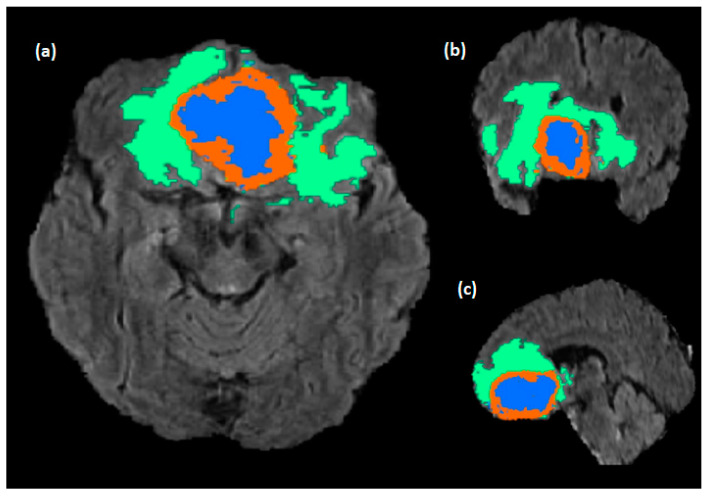
Brain tumor sub-region from the RSNA BraTS2021 dataset (BraTS2021_00318). The NCR/NET, ET, and ED regions are highlighted in blue, red, and green, respectively. Where (**a**–**c**) are the Axial, Coronal, and Sagittal slices.

Glioblastomas (GBM) are the major brain tumors’ category, and from a clinical perspective, they exhibit a complex aggressiveness level, and they counter inter-expert delineation uncertainties. A Magnetic Resonance Image (MRI) is the dominant tool for analyzing medical images of the brain and it provides an accurate imaging diagnostic along different modalities with different protocols and configurations. Conventional MRIs involve using four sequences named T1-weighted (T1w), T1-weighted with gadolinium contrast (T1w-gd), T2-weighted (T2w), and a fluid suppression sequence called fluid attenuated inversion recovery (FLAIR). Glioblastomas typically display a necrotic center, an abnormal area with contrast enhancement, and a peritumoral region with a high FLAIR signal, which can indicate edema, tumor infiltration, or a combination of both. A common issue with segmenting medical images is the long-time process needed from radiologists and labor-intensive process. Additionally, manual segmentation is prone to human error and variability in inter and intra-reader intra-qualitative visual evaluation among radiologists, which can lead to inconsistent or inaccurate results when different radiologists perform the task. Brain tumors consist of three main sub-regions; this adds complexity to the segmentation procedure where some sub-regions, like enhanced tumors (ET), are more complicated than other regions due to the interventions of tumorous and healthy tissues and other classes (Figure 1). Precise brain tumor segmentation is considered a big challenge due to the intricacy of tumorous tissues and it is critical in precision patient care. The need for a precise identification of tumor sub-regions from MRI takes place during the monitoring of tumor growth within the long-term treatment process as well as the treatment planning, generating the radiotherapy maps and helping clinicians to precisely target the tumorous tissues while protecting the healthy ones. A comprehensive survey was conducted by Liu et al. [1] about the highlighted deep learning methods used for brain tumor segmentation. The most common challenge of medical imaging is the lack of annotated images produced by experts, therefore many data augmentation methodologies have been proposed. Nalepa et al. [2] examined the technical aspects and effects of various data augmentation techniques in the context of brain tumor segmentation. Therefore, an automatic sub-region segmentation is an alternative for this tedious work and can help to overcome these issues by providing more consistent and accurate results, reducing biases and variabilities, and increasing the efficiency of the diagnostic process.

For activating such efficient auto-segmentation and delineation of brain tumors, the Multi-modal Brain Tumor Segmentation Challenge (BraTS) has been provided to enable deep learning common platforms to compete using the common multiparametric MRI of gliomas. Ghaffari et al. [3] have analyzed the state-of-the art performance of BraTS 2012–2018 datasets and have studied the parameters that affect the performance of different deep learning models used for brain tumor segmentation. 

The main contributions of this work are as follows:

- Proposing a new U-Net-based architecture for brain tumor segmentation called “Bridged U-Net-ASPP-EVO” with its two variants.
- A comparative experimental work between five state-of-the-art models is conducted for qualitative validation.
- Providing ablation studies of the basic optimization schemes (optimizers, loss functions, hyperparameters) based on experimental work to emphasize their impact on the overall segmentation performance.

The rest of the paper is arranged accordingly; the related works are illustrated in Section 2. Section 3 describes materials and methods used. Section 4 includes the proposed work; Section 5 demonstrates the experimental study. Section 6 represents the Results. Discussion is illustrated in Section 7. And finally, the conclusion and future directions are explained in Section 8.

## 2. Related Work

Recently, medical image application in general and segmentation in particular has become vastly dependent on deep learning due to its trusted and qualitative results. For instance, the novel proposed architecture of U-Net in 2015 [4], based on encoder–decoder architecture, that consists of two major parts, the first being the contracting path where training and compressing the input data is achieved through Convolutional Neural Networks (CNN), and the second being the expansion path where the decompression of the knowledge feature maps is done to retrieve the input data images resolution for comparing with the ground truth. U-Net architecture has witnessed tremendous optimization models that have achieved better results than the basic model; instances of this for the U-Net-based models include Res U-net [5,6], Dense U-Net [7,8], and attention U-Net [9], which have been widely employed for the segmentation of brain tumors. 

Furthermore, blocks like residuals, attention gates, and dense blocks were used extensively to produce new ensembled models that are built upon each other’s models in an incremental fashion to improve the score of segmentation. Another approach of deep learning that uses generative adversarial learning is the vox2vox model [10], in which a U-Net architecture model has been used in the generator and a DCNN model has been used at the discriminator network; another generative adversarial-based model that uses U-Net architecture was used by Peiris et al. [11] and their model involves three modules, the first one is called segmentation network and it is a U-Net architecture model, the second is the Critic module, which consists of a fully convolutional adversarial network that depicts a Markovian PatchGAN, and finally a Virtual Adversarial Training (VAT) block used for generating the fake adversarial samples to help the segmentation network in avoiding false predictions on new samples. Overall, the generative adversarial approach requires more computational power and training time due to the usage of multi-network architectures (generator and discriminator) which in turn leads to more parameters. On the other hand, the U-Net architecture has also witnessed numerous major modifications to produce variety in the newly shaped architectures such as U-Net++ [12] and Separable 3D (S3D) U-Net [13], which utilize separable U-Net networks together. The deep learning approaches used for brain tumor segmentation are usually divided into single or multiple path architecture or encoder–decoder structures, where basically single and multiple structures are used to extract features and classify the pixels of the input image patch. On the other hand, the encoder–decoder network architecture is designed to perform end-to-end tumor segmentation, where the encoder is responsible for the feature extraction and the decoder performs feature-to-segmentation mapping. The single path networks are mainly used for efficient computations [14,15]. However, using multi-path networks to help in extracting different features with different scales. Havaei et al. [16] have used two pathway networks for learning both local and global contextual tumor information, while Castillo et al. [17] have used three pathway CNN architectures for segmenting brain tumors. However, both single and multiple path CNN architectures use different kernels sizes, and it is highly dependent on the input patch size quality and size, where the small patches involve a lack in spatial information, and the large size patches require more computational power. Another drawback of these models’ structures is that the fully connected layer FCN used to perform the feature-to-segmentation output cannot represent the entire feature space efficiently and cannot perform end-to-end segmentation and more sophisticated FCNs might overload the GPU’s memory. Encoder–decoder architecture was proposed to tackle the problems with the previous models and to perform the segmentation tasks more efficiently in an end-to-end fashion. Such encoder–decoder models have become a major area of research, especially U-Net-based models due to their efficient performance compared with the other models mentioned before.

## 3. Materials and Methods

**Figure 2:**
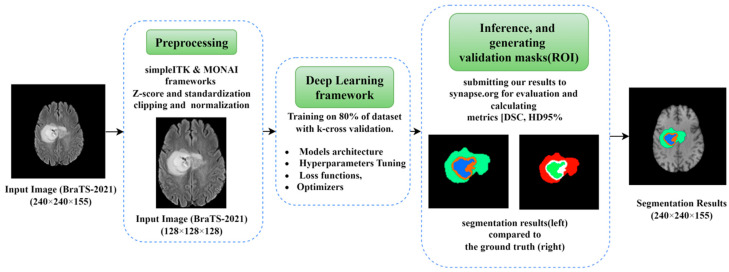
Graphical methodology of optimization techniques used for brain tumor segmentation.

A graphical structure of our complete work in brain tumor segmentation using optimization perspectives of DLs is shown in Figure 2. The workflow of this paper is divided into three main parts; the first part is the preprocessing, which includes the methods and frameworks applied on the raw dataset images before being fed into the network. The second part is the core deep learning framework, which includes the architecture along with all the parameters and hyperparameters used within. The third part includes the inference and generating the segmentation results for the validation dataset. 

Our work consists of performing brain tumor segmentation with different schemes of optimization; such optimization schemes are as follows: 

1. Model architecture: Different DL architectures created on U-Net have been used and evaluated against the segmentation. The applied models include 3D U-Net, modified U-Net, attention U-Net, Residual U-Net, attention Residual U-Net, Recurrent Residual U-Net (R2AU-Net) [18], and our proposed model.

2. Optimizers: In this work, three main optimizers were used for validating the results and providing a comprehensive experimental finding including Adam (Adaptive Moment Estimation) [19], which was extensively used for deep learning models. Another variant of Adam optimizer is called AdamW [20], which uses the weight decay parameters, Ranger 20 and Ranger21 [21,22], and was built on an Adam optimizer. Generally, Ranger optimizer provides a more generalized and stable method for small batch size and noisy labels [23]. 

3. Hyperparameters tuning: Huge volumes of the literature were focused on hyperparameter tuning, since deep learning was and still is an empirical field in which hyperparameters still affect the performance of the CNN architectures. Different hyperparameters were experimented with, such as epochs, batch size, learning rate, activation function, and sliding window sizes of the region of interest, and the ones that achieved better results were used in the final model after observing the performance. 

4. Loss functions: We have experimented with various loss functions depending on the evaluation metrics used. Choosing loss functions may cause more time to train the model, for example using Hausdorff Distance loss has caused very slow training, therefore we did not consider it in this study. The major loss functions used in this work are dice loss, dice and cross entropy loss “Dice_ce”, focal loss, dice and focal loss “Dice_focal”, and dice and boundary loss “Dice boundary”. 

### 3.1. Datasets

**Figure 3:**
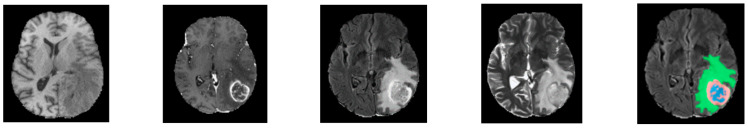
Axial slice representation of (BraTS2021_01386) sample of the four modalities from left to right (t1, t1-ce, FLAIR, t2, and the corresponded segmentation labels for the 3 tumor classes on the FLAIR image. Green: peritumoral edematous/invaded tissue, Red: GC enhancing tumor, and Blue: necrotic tumor core taken from ground truth.

In this paper, we have applied our DL model to datasets from MICCAI challenge (BraTS-2020 and BraTS-2021). The RSNA-ASNR-MICCAI BraTS 2021 dataset [24] is constructed as follows: 2040 data samples in total, 1251 samples are considered for training, 219 samples are used for validation through the synapse.org. Each data sample consists of four 3D MRI sequences related to one patient (T1, T1-gd, T2, and T2-FLAIR). All the multiparametric mp-MRI are acquired using different protocols and scanners from multiple institutes of an isotropic voxel volume (1 × 1 × 1 mm^3^); each 3D-MRI modality has a size of 240 × 240 × 155. For the training dataset, the ground truth(mask) was annotated, delineated, and segmented by expert radiologists to provide e-subregions of tumors (necrotic and non-enhancing tumor “tumor core TC” as label-1, the peritumoral edema “whole tumor WT” as label-2, and the enhancing tumor “ET” as label-4). Each tumor subregion is responsible for describing the tumor behavior and properties, e.g., ET shows the hyper-intense signal in the T1-Gd modality, while the non-enhancing tumor (NET) and the Necrotic Tumor (NCR) are considered as hypo-intense in T1-Gd modality when comparing with the T1 modality. Both NET and NCR represent the tumor core (TC) subregion, and finally the whole tumor (WT) that describes the full extension and size of the tumor is constructed from the Peritumoral Edema (ED) and the tumor core TC. Table 1 shows the used datasets input image size and number of samples in both validation and training datasets. A BraTS-2021 sample MRI with all modalities from training dataset, in addition to the provided mask, is pictured in Figure 3.

### 3.2. Experimental Setup and Configurations

In general, medical image analysis requires high system hardware configuration because, usually, medical image datasets come with big image sizes. Moreover, deep learning models are computationally costly; a compromised medical data resolution is especially not preferred to overcome the processing power needed. Our experiments were done using an Ubuntu 20.04, with 197 GB of RAM and Nvidia RTX A6000 of 48 GB memory. Our configuration of Python 3.10 was cuda version 11.7 and Pytorch 1.12.1. 

### 3.3. Preprocessing

MRI sequences are acquired through different configurations, where each MRI has four volumetric channels related to MRI sequences mentioned above. And the labels (WT, ET, CT) represent the regions of tumor by following the same cropping protocol of volumes to be (128 × 128 × 128) for height, width, and slices. For observing the performance of our model while training, we have split dataset into 80% (1000 samples) for training and 20% (251 samples) for validation. Medical Open Network for Artificial Intelligence (MONAI) [25] and simpleITK [26] frameworks were used for preprocessing MRI images and for basic data augmentation (e.g., random flip, random shift, random contrast intensity adjustment, random rotation of 90 degree, random Gaussian noise and smoothing, and z-score for scaling and standardization) to avoid overfitting.

Instance normalization was used as the main normalization algorithm while training of all used deep learning models was used for this work, except for the proposed model [27]. An instance normalization layer was used for the input images. For example, for an input image X ϵ R (B × C × H × W) where B, C, H, and W are the batch size, channels, height, and width, respectively, then, the instance normalization of an affine transformation with gamma and beta is given by:

(1) $$\mathrm{IN}\left(X\right)=\;\gamma \frac{X-\underset{H,W}{E}\left[X\right]}{\sqrt{\underset{H,W}{\mathrm{Var}}\left[X\right]+\varepsilon }}+\beta$$

where E is the mean, ε is used for the numerical stability, and the affine transformation is used to scale or shift the normalized result. The features in the input in mage is dependent on the number of channels.

### 3.4. Ranger Optimizer and Gradient Centralization (CG)

For quite a long time, Adam optimizer used to be the dominant optimizer for deep learning applications for image segmentation. But afterwards, Ranger optimizer [28] was evolved from combining both a Rectified Adam optimizer and Lookahead optimizer [29], which was developed based on the neural network loss surfaces to enhance the deep learning stability in terms of training and speeding up the convergence. Both optimizers were combined into one synthetic optimizer, called Ranger, which performed better than any optimizer when used alone. The benefit of using Lookahead optimization algorithm is the interpolation between two sets of weights (fast and slow), where the slower weights help in maintaining longer term stability, while the faster weights enable the “look ahead” feature that allows the pre-exploration mechanism of the surface loss that achieves faster convergence. Adaptive learning rate in Rectified Adam optimizer is achieved using rectifier function based on the actual variance encountered. Therefore, RAdam is more robust with learning rate variations. In our experiment, an initial learning rate was set to (η = 0.0003), and it was reduced to half of its value after 100 epochs (η = 0.00015) using the flat-cosine weight–decay method used for weight averaging:

(2) $$\eta_{i}=\frac{1}{2}\left(1+\cos\left(\frac{i\pi }{E}\right)\right){*\eta }_{\mathrm{start}}$$

where E is the total number of epochs, ƞ_i_ is the learning rate during the ith epoch of training, and ƞ_start_ is the starting learning rate.

Moreover, Ranger optimizer uses Gradient Centralization (GC) [30], which is a generalized gradient descent with constrained loss function that centralizes the gradients’ vectors with zero mean. Ranger applies the GC optimization technique on all Conv layers and FC layer of U-Net as well. It was discovered that employing GC makes training more effective and stable. Though for each layer, the gradient’s mean is calculated and subtracted along the first dimensional axis. The centralized gradient is given by the formula:

(3) $$G_{\mathrm{center}\left(t\right)}=\nabla f_{t}\left(\theta_{t-1}\right)-\mu \left(\nabla f_{t}\left(\theta_{t-1}\right)\right)$$

where µ is the mean. GC forces the regularization and constraint on loss function that smooths the optimization scheme, where a smoother training curve and faster convergence were achieved when using GC.

Results show that Ranger-2020 has achieved better results than Adam optimizer, and Ranger-21 has outperformed both with a slight improvement in DSC and Hausdorff distance too.

### 3.5. Loss Functions

In DNN, loss function is a critical choice while performing the optimization. Choosing the right loss function determines the overall performance of DNN applications because it emphasizes the error measurement between the ground truth and the predicted segments. Cross-Entropy is a public choice for general applications of deep learning, but for segmentation, dice loss is a dominant choice. We have experimented with different loss functions. Loss functions are commonly used relatively with the metrics; for instance, since the common metrics used to evaluate brain tumor segmentations are Dice Similarity Score (DSC) and the Hausdorff Distance 95 percentile (HD95%), then loss function is to be determined accordingly. 

Inspired from the commonly used loss “Dice Loss” [31], we have chosen it to be our main loss function due to many reasons, such as the good performance, the compatibility with the DSC, and the faster training criterion. Dice loss is defined by this formula:

(4) $${ℒ}_{\mathrm{dice}}=1-\frac{1}{N}\underset{i}{\sum }\frac{(p_{i}{*g}_{i})+\varepsilon }{p_{i}^{2}+g_{i}^{2}+\varepsilon }=1-\frac{2\times \sum_{i}^{N}p_{i}{*g}_{i}+\varepsilon }{\sum_{i}^{N}p_{i}^{2}+\sum_{i}^{N}g_{i}^{2}+\varepsilon }=1-\mathrm{DSC}$$

where N is the number of voxels and p_i_ and g_i_ are the output ROI voxels and the ground truth voxels of the brain tumor mask, respectively. ε is a smoothing factor used to avoid the zero division (set to ε = 10^−5^ in our experiment). Dice loss achieves good quality in terms of establishing the balance between the ROI and the background by tuning the weights matrices. 

Another identical loss function is called the Jaccard loss, and it can be derived from dice loss. Choosing the loss function could be derived from the metrics used for evaluation. Hausdorff loss was used but the main drawback of this loss is the time consumed while training due to the calculations needed. The dice loss and cross entropy loss compute the weighted sum of these two losses. The cross-entropy loss is given by the following:

(5) $${ℒ}_{\mathrm{CE}}\left(p,g\right)=-\overset{N}{\underset{i=1}{\sum }}\overset{L}{\underset{l=1}{\sum }}g_{i,l}\log\left(p_{i,l}\right)$$

Here, N is the number of voxels and L is the number of classes = 3, p_i,l_ is the predicted probability map, and g_i,l_ is the discrete ground-truth probability map. The dice-cross entropy loss is the sum of both losses, and it is given by the following:

(6) $${ℒ}_{\mathrm{dice}-\mathrm{CE}}={ℒ}_{\mathrm{dice}}+{ℒ}_{\mathrm{CE}}$$

Other loss functions used are focal loss, dice-focal loss, and dice boundary loss. 

Ma J et al. [32] have provided a comprehensive overview of various loss functions used for medical image segmentation; we have tested the dice-focal loss function but dice loss and dice-cross-entropy (Dice-CE) losses have outperformed the dice-focal loss.

## 4. Proposed Work

The main proposed model used for this work is centered on the 3D U-Net with major modifications; it is called the Bridged_U-Net ASPP_EVO and it consists of four levels of an encoder–decoder U-shaped model. This model involves using the followings attributes (blocks):

### 4.1. Atrous Spatial Separable Pyramid ASPP Block

**Figure 4:**
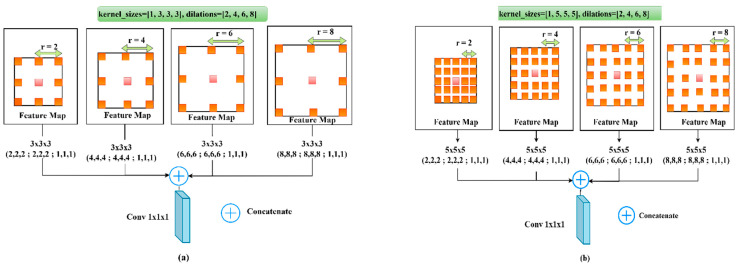
ASPP, Atrous Spatial Pyramid Pooling and convolution block. (**a**) Using [1, 3, 3, 3] kernel size, (**b**) ASPP using kernel size [1, 5, 5, 5].

The ASPP method was designed for capturing the multiscale contextual information between encoder and decoder used specifically for semantic segmentation [33,34]. However, instead of using a basic convolution (usually rate = 1) and Max-Pooling or Average-Pooling in the blockage of the 3D-U-Net, we have used the ASPP module with different rates (2, 4, 6, 8) instead of depth-wise and point-wise convolutions (basic convolution) to reduce the computation complexity. The formula of the ASPP module is given by the following:

(7) $$y\left[i\right]=\underset{k}{\sum }x\left[i+r.k\right]w\left[k\right]$$

where the stride needed to sample the input feature map is represented by r, which is the rate connected to it. X and w are the input signal and filter, respectively. When r = 1, the fundamental standard convolution is a particular instance of the Atrous separable convolution. It is obvious that the Atrous convolution enables kernels at any deep CNN to have a wider field of view. Our ASPP block is shown in Figure 4. It supports the small field-of-view for the accurate localization of ROI and the context absorption without increasing the parameters and computation due to the zeros introduced between filter values.

ASPP block is used at the bottleneck of the network with two sets of dilations and two sets of convolution kernel-sizes (Figure 4). (ASPP) was implemented in the bottleneck to capture the multi-scale features of various tumor sizes. The details of the ASPP block used have four dilation rates [2, 4, 6, 8] with [1, 5, 5, 5] kernel sizes, which are concatenated using (1 × 1 × 1 normal Convolution).

### 4.2. Evolving Normalization Activation Layer (EVO_NORM)

Another normalization scheme is used while building the 3D U-Net blocks for each layer, which is EvoNorm, the Evolving-Normalization activation layer [35], in which several heuristics are used for designing normalization layers and activation layers in order to optimize the building blocks of the CNN architecture and their performance, thus preventing overfitting. Evolving Normalization or Evo norm is used within the 3D-convolutional blocks at both paths of U-Net; moreover, the pooling operations performed while down-sampling the feature maps at the encoder is a concatenation of max and average pooling, which helps in reducing the information loss. The EvoNorm-S series is batch-independent and it refers to the sample-based layers. The mathematical formula of *EvoNorm-S*0, which is used in the building blocks, is given by the following:

(8) $$EvoNorm-S0=\frac{x\sigma \left(\upsilon_{1}x\right)}{\sqrt{s_{w,h,c/g}^{2}\left(x\right)}}\gamma +\beta$$

where *w,h,c*/*g* refers to the width, height, and channels of the input tensor (*x*) and ./*g* refers to the aggregation performed in group fashion. γ and β are the affine transformation parameters and σ refers to the standard deviation.

### 4.3. Squeeze and Excitation with Residual Block (SE-Block) 

**Figure 5:**
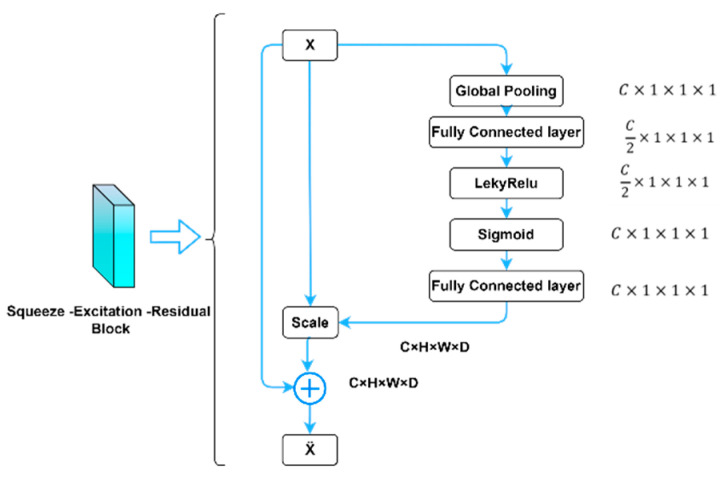
Squeeze–Excitation–Residual Block.

SE-block [36] pictured in Figure 5 focuses on important feature maps and suppress the less important ones. This improves the model’s ability to identify the target object in the input image, which can lead to better segmentation results. SE-Block is added after the three dilations of convolutions with two activations are used leakyReLU and then the sigmoid activation function.

### 4.4. Bridge Layer 

**Figure 6:**
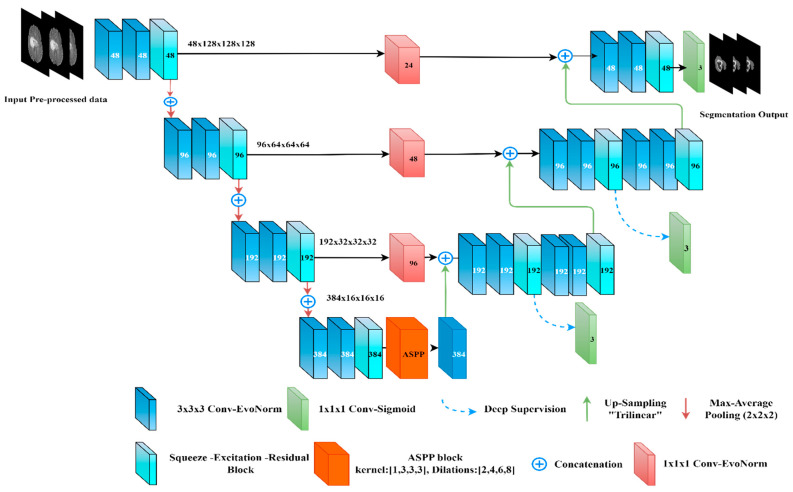
Our proposed model “Bridged-U-Net-ASPP-EVO (variant-1)”. Feature maps are shown at the encoder side; channels number is shown at each block.

This layer is used between the encoder–decoder levels using a (1 × 1 × 1 Convolution with Evo Norm layer). Considering the use of a half channel number during convolution, it is used to map the low-level features of the encoder before concatenating the resultant feature map with the next high-level decoder features. This is used to diminish the semantic gap between the low-level features and the high-level ones [37], and to preserve the spatial information from the encoder. To maintain the matching between the bridge blocks, a (1 × 1 × 1 Convolution) followed by trilinear interpolation is used within the up-sampling layers as shown in Figure 6.

**Figure 7:**
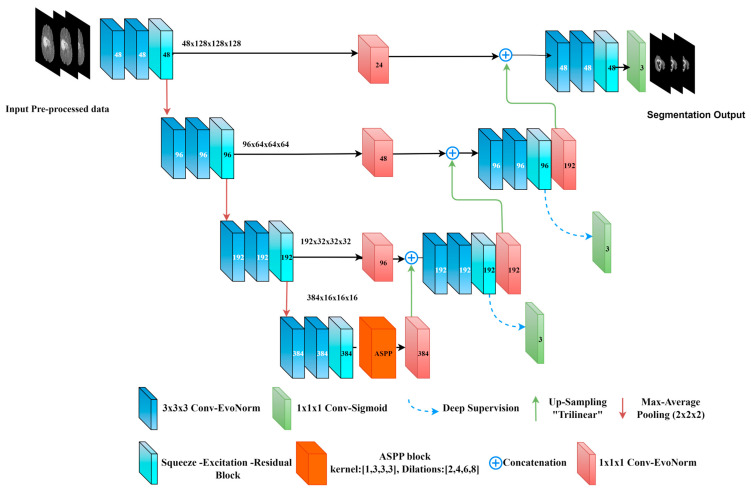
Our proposed model “Bridged-U-Net-ASPP-EVO (variant-2)”. Feature maps are shown at the encoder side; channel number is shown at each block.

Deep supervision was used while training at all decoder levels except the bottleneck level. Following the same concept of basic U-Net, a (1 × 1 × 1 Convolution) kernel with three channels is used with a sigmoid activation function before the final output. Moreover, we have modified the proposed model (the decoder side only) where (3 × 3 × 3 Conv) with EvoNorm was used instead of the two dilated convolutions and SE-Block. We called the resultant model “Bridged U-Net-ASPP-EVO variant 2”. Furthermore, the ASPP model shown in Figure 4a was used, which involves using [1, 3, 3, 3] kernel size instead of the [1, 5, 5, 5], which was used for the proposed model variant 1. This model is shown in Figure 7. 

## 5. Experiments

Inspired from other state-of-the-art models that have used U-Net-like architecture, we have designed an ensembled network inspired from the previous networks for the BraTS 2020 challenge. Multiple experiments have been done following different levels of optimizations. In order to evaluate our model (both variants) with other state of the art models, we have considered using four state-of-the-art models as follows:

1. 3D U-Net: This architecture was proposed for the BraTS-2020 challenge [38]. The network architecture consists of four levels of encode–decoder; leakyRelu is used as the activation function; group normalization was used along each convolution; and auxiliary segmentation output from the ground truth is used for deep supervision used at the decoder side. For the proposed model with minor modification, see Figure 4.
2. Attention U-Net: The second U-Net architecture is also inspired from the BraTS-2020 challenge, and it is based on the 3D_U-Net mentioned above; the concatenation between the encoder and the decoder uses an attention gate.
3. R2_Attention U-Net: Recurrent Residual Attention U-Net was anticipated in [18] for multimodal medical images’ segmentation. The same proposed architecture was used for our experiment.
4. Modified U-Net: based on the 3D U-Net, the architecture was modified by adding one more convolution block level-wise. The other network configurations remained the same (LeakyRelu activation function and group normalization, in addition to deep supervision).

### 5.1. Post Processing

This phase was implemented using the thresholding of the generated segmentation map [39]. For constructing the segmentation map, the MRI original labels (NCR/NET, ED, and ET) were used instead of the 3-channel volume (label-1 refers to TC, label-2 refers to Ed or WT, and label-4 refers to ET) by using Boolean functions. Eventually, corresponded voxels below 20 voxels of the three labels are ignored. And, since the ET label is a sensitive and complex region of the tumor, its voxels below 300 voxels were replaced with NCR/NET to verify that those voxels are kept considered as a portion of the tumor’s core. 

### 5.2. Evaluation Metrics 

For brain tumor segmentation, the considered regions are the ET, TC, and WT, where the prediction results of the segmentation will be evaluated in these three regions. The evaluation metrics differ from one application or task of deep learning to another; for brain tumor segmentation, the most suitable metrics are the dice score and the dice similarity coefficient (DSC) and the Hausdorff distance.

#### 5.2.1. Dice Similarity Coefficient (DSC)

Dice score [40] is a measurement of overlapping areas between the predicted results and the ground truth. DSC is defined as follows:

(9) $$\mathrm{DICE}=\frac{2\left|S_{g}\cap^{\;}S_{p}\right|}{\left|S_{g}\right|+\left|S_{p}\right|}=\frac{2\mathrm{TP}}{2\mathrm{TP}+\mathrm{FP}+\mathrm{FN}}\;,\in \left[0,\;1\right]$$

where S_g_ and S_p_ are the area ‘number of pixels’ of the ground-truth mask and the predicted mask, respectively. TP, FP, and FN are the true positive, false positive, and false negative, respectively. In general, since brain tumor tissues come with complex structures, a specific class (e.g., ET) can not only be contained in one region, but it can be formulated through separate regions (group of pixels), which makes the overlapping measures tricky and intractable. 

Overlapping measures for big objects is well-suited, but for small regions and multi-region classes, DSC will not be the perfect metric, although another segmentation evaluation metric will be used.

#### 5.2.2. Hausdorff Distance95% (HD95%)

Hausdorff distance [41,42] is a distance-based evaluation metric used for segmentation; it consists of measuring the distance between two regions or subsets.

For brain tumor evaluation, Hausdorff distance (HD95%) became an official metric used for evaluation and it determines the distance between the ground-truth region and the prediction of the segmented area. The average HD between two finite subsets X and Y is given as follows:

(10) $$d_{\mathrm{AHD}}\left(X,Y\right)=\left(\frac{1}{X}\underset{x\in X}{\sum }\underset{y\in Y}{\min}d\left(x,y\right)+\frac{1}{Y}\underset{y\in Y}{\sum }\underset{x\in X}{\min}d\left(x,y\right)\right)/2$$

Hausdorff distance is measured in millimeters or voxels. The equation above can be written as follows:

(11) $${\mathrm{HD}}_{\mathrm{avg}}=\left(\frac{G\;\mathrm{to}\;S}{G}+\frac{S\;\mathrm{to}\;G}{s}\right)/2$$

where G is the ground truth and S is the segmentation result.

Other used metrics for brain tumor segmentation are sensitivity and specificity, where sensitivity measures the proportion of actual tumors that were correctly identified by the algorithm. A higher sensitivity value indicates that the algorithm has a better ability to detect tumors. Sensitivity is given by the following:

(12) $$\mathrm{Sensitivity}=\frac{\mathrm{True}\;\mathrm{Positive}}{\mathrm{True}\;\mathrm{Positive}+\mathrm{False}\;\mathrm{Negative}}$$

while the specificity measures the proportion of non-tumor regions that were correctly identified by the algorithm. A higher specificity value indicates that the algorithm has a better ability to correctly identify non-tumor regions. Specificity is given by the following:

(13) $$\mathrm{Specificity}=\frac{\mathrm{True}\;\mathrm{Negative}}{\mathrm{True}\;\mathrm{Negative}+\mathrm{False}\;\mathrm{Positive}}$$

The preceding evaluation metrics are the major metrics used for medical image segmentation, where these metrics are also considered by the challenge website.

## 6. Results

The BraTS dataset challenge uses the ‘ranking-then-aggregating’ aspect in which the evaluation in general is based on two different metrics (DSC and HD95%). As a result, there will be six ranks for each test case (one for each segmented region “TC, ET, WT”) and then all the ranks are averaged among all test cases for the final normalized rank. We have done the experimental work on BraTS-2020 and BraTS-2020 and it was noticed that the results for both datasets were coherent due to the standardized protocol followed for both the challenges and the un-biased annotated data, except that the results of the segmentation scored better metrics for BraTS-2021, due to the size of the dataset (1251 example compared with 369 examples in BraTS-2020). The enhanced tumor (ET) region scored the lowest scores in all cases. Our proposed model of Bridged U-Net that emphasizes Atrous Spatial Separable Convolution and pooling used to capture multi-scale ROIs has achieved the results of DSC and HD95 shown in Table 6, provided by the evaluation platform. Beyond the training phase, in order to generate the segmentation prediction inferences, Test Time Augmentation (TTA) was used before the validation and evaluation stage to enhance the segmentation output.

### 6.1. BraTS 2020 Segmentation Results

In order to generate a valid workflow of our experimental results, we have considered both BraTS-2020 using the challenge submission portal (https://ipp.cbica.upenn.edu/ (accessed on 10 November 2020)) and the BraTS-2021(https://www.synapse.org/ (accessed on 10 December 2021)) dataset for validation. Initial experiments were performed on the BraTS-2020 dataset and then the same experiments were performed on the BraTS-2021 challenge dataset.

**Figure 8:**
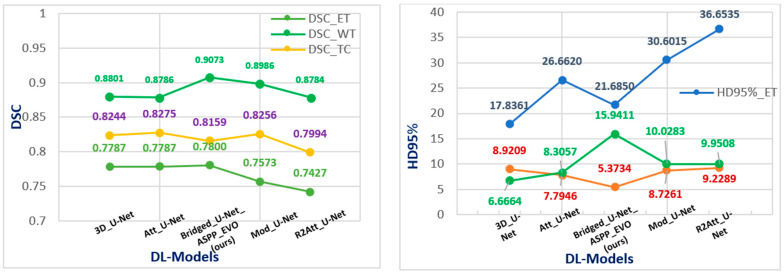
Segmentation performance of different U-Net architecture results based on DSC (**left**) and HD95% (**right**) on BraTS 2020 dataset.

Firstly, we have performed our models and optimization configurations against BraTS 2020 and evaluated our segmentation results on the validation dataset (125 sample). The five models’ performance according to the BraTS-2020 challenge is shown in Figure 8, noting that the Bridged_U-Net_ASPP_EVO (model-5) v1 is not used where the modified U-Net is used (Att-U-Net is the attention_U-Net, mod_U-Net refers to the modified U-Net, and R2Att_U-Net stands for Recurrent Residual Attention U-Net). The configuration used to get an un-biased model-based performance comparison are detailed in Table 2. These configurations were considered after many experiments, as is explained in Table 3.

### 6.2. Ablation Study of Sliding Window Impact

**Figure 9:**
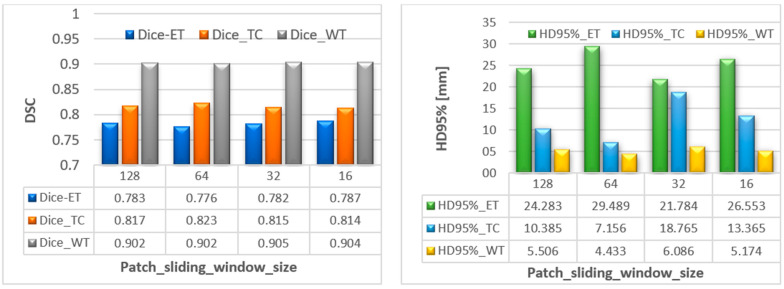
Segmentation results of patch sliding-window-based performance for our proposed model variant 1. Dice score on left and HD95% on right (“*x*-axis represents the experimented sliding-window size”).

Another experiment was performed to check the effect of patch sliding window size to check if it does affect the segmentation results, since brain tumors sizes are different within dataset examples. The results of applying four different sliding window sizes ([128 128 128], [64 64 64], [32 32 32], [16 16 16]) is shown in Figure 9. Although a slight difference of the effect of sliding window size on dice score is noticed, it shows a clear difference when corresponded with Hausdorff distance; therefore, considering the minimum average provided, we decided to choose a sliding window size of [128, 128, 128]. We have conducted multiple similar experiments to determine other hyperparameters. The obtained results have contributed towards better performance and the final outcomes possessed the configurations used in Table 2.

### 6.3. BraTS 2021 Segmentation Results

Similarly, the same experiments were conducted on a BraTS 2021 validation dataset (219 cases) Through the experiments, it was found that a modified U-Net is time and memory consuming, though we did not use this model for the BraTS 2021 dataset, but we have used the Bridged U-Net ASPP EVO variant 2 (Figure 7) instead and it has achieved the state-of-the-art results. Also, different hyperparameters like learning rate, batch size, and activation functions were implemented through the experiments but due to the minor impact on the overall performance we did not consider including related ablation studies in this work. The final configurations are detailed in Table 2.

### 6.4. Ablation Study

To analyze the impact of optimizers shown in Table 4 and loss function shown in Table 5 on the overall performance of DSC on the BraTS-2021 validation dataset, we have conducted a series of experiments which involves multiple optimizers and loss functions only to our proposed model variant 1. 

Table 6 shows the results of similar experiments conducted on BraTS 2021 validation dataset (219 cases). K-cross validation (k = 5) was applied for the Bridged_U-Net ASPP_EVO v2 for generalization and the results shown in Table 7 are slightly differed from results of a single fold-training. Therefore, we did not perform K-cross validation on all the other models because it will be time consuming. We included the number of parameters of each model along with the training time; however, the inference time needed for generating ROIs for single sample from the validation dataset was in a small range of variation between the five models within range of [1.1–2 s].

### 6.5. Performance Comparison

We provide an experimental analysis of our proposed model and a qualitative comparison (both variants) with other recent state-of-the-art models based on U-Net architecture results on the validation data only applied on the BraTS 2020, and BraTS 2021 validation datasets in terms of dice similarity coefficients (DSC). The experimental performance comparison is presented in Table 6; in Table 8, we list the highlighted features of the experimented state-of-the-art models and common limitations found in the literature compared with our proposed model. In Table 9, we compare the performance of our model to other models found in the literature. 

## 7. Discussion

**Figure 10:**
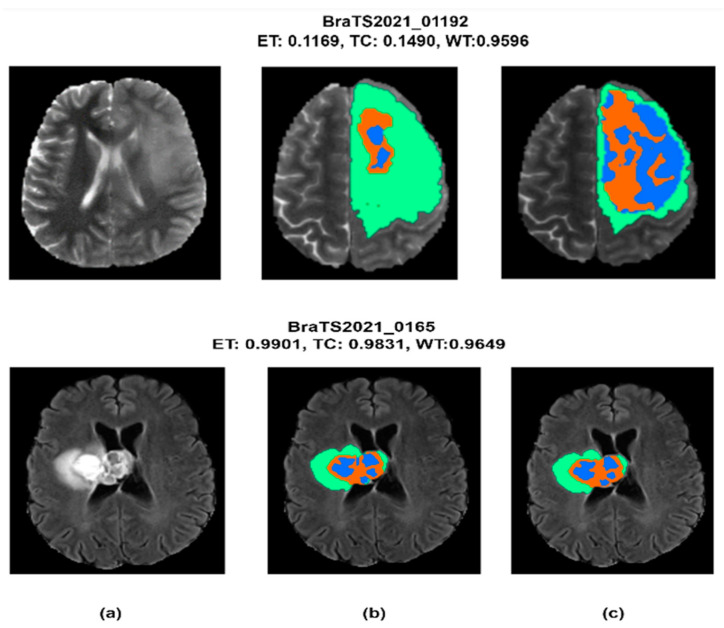
Segmentation results on the local BraTS-2021validation dataset along with MRI_id, (**a**) FLAIR MRI, (**b**) ground truth segmentation mask, (**c**) our model segmentation results. The top image represents a bad prediction while the bottom image represents an excellent prediction.

**Figure 11:**
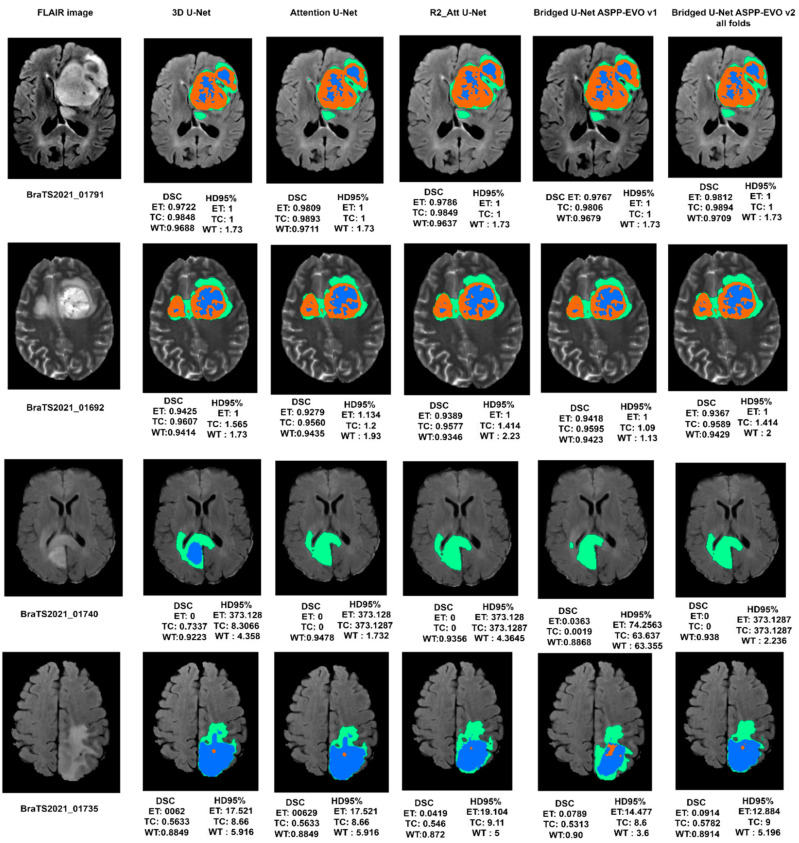
Visual segmentation results of the experimental DL models along with the proposed model on BraTS 2021 validation dataset.

A generalized and optimal experimental approach was conducted to overview U-Net models for brain tumor segmentation. Different optimization schemes were applied to find the best combination. Standard data like the BraTS-2020 & 2021 challenge was used to evaluate models with two metrics (DSC and HD95%). It is clear from Table 6 that our proposed Bridged_U-Net ASPP_EVO *v*1 and Bridged_U-Net ASPP_EVO *v*2 have improved the overall segmentation results according to the mean DSC. The main improvement was made against the enhanced tumor class (ET) DSC and HD95% results when using the Bridged_U-Net ASPP_EVO *v*1, and this was due to using a larger kernel of the ASPP block [1, 5, 5, 5]. Figure 10 shows good and bad inferences of the local validation dataset along with the ground truth while Figure 11 shows some results of our Bridged U-Net_ASPP_EVO_v1 on the validation dataset with an excellent segmentation result along with bad segmentation results related to different data discrepancies like the absence of one of the tumor labels’ sub-regions, though if the submission assigns false positive voxels in such cases, then the BraTS evaluation protocol will assign the worst possible value for both metrics (DSC = 0, HD95% = 373.13) while a correct prediction of empty labeled ROIs will yield to best values (DSC = 1, HD95% = 0). Scores are acquired after the submission to the challenge (BraTS 2021 validation dataset) of an average overall performance shown in Table 6 where the top two rows of Figure 11 represent good results, and the bottom two rows represent bad segmentations. We noticed that all of the implemented models in this work have resulted in bad performance for 10 cases out of a total of 219 cases, where these 10 cases have suffered from the absence of one or two tumor sub regions (ET and TC). These cases are [BraTS2021_ 01729, 01731, 01738, 01739, 01740, 01741, 01743,01747, 01774, and 01784]. Where these ten cases have DSC = 0, this affects the overall average dice score of all models. Luu et al. [49] (who *slightly outperformed our proposed model*) have used the nnU-Net approach, which was found to be very time consuming due to the extensive fine tuning DL components (hyperparameters, optimizers, activations, etc.) [50,51,52,53,54,55,56,57,58,59,60]; moreover, recent optimizers and loss functions need to be implemented manually because these two elements are fixed in their original model. Moreover, the proposed model can be efficiently used for other applications of deep learning in medical image segmentation other than in brain tumors.

### Limitations and Challenges

The main challenges in this work were the vast number of optimization parameters and schemes that were done empirically to deliver our last blueprint criteria. Another challenge is the high degree of computational power to perform the training that is required, which still needs to be considered, although we have reduced the network trainable parameters to fit with the hardware.

## 8. Conclusions and Future Work

Deep learning became an indispensable tool for medical image segmentation, and it continues to improve its performance and accuracy. However, the major concern is directed towards optimizing deep learning, which includes multi-level optimization such as optimizing the deep network architectures, performing ensembled learning, hyperparameter tuning (empirical approach), and choosing the more efficient optimizers and loss functions. In this paper, we have presented an experimental approach to optimization to provide a fingerprint of the best deep learning practical pathologies for brain tumor segmentation. We have proposed a modified Bridged U-Net architecture with an evolving normalization layer and Atrous Spatial Pyramid Pooling (ASPP), which has proven to be efficient, and it outperformed other state-of-the-art models. Furthermore, we have experimented with different optimization criteria to provide an overview of how deep learning tools are still empirical and need to be adjusted; we have conducted ablation studies to define the best combinations of deep learning blocks (optimizers and loss functions). We hypothesized that over-optimization could reach a level where no improvements could be provided. Moreover, it was found that simple base models like 3D U-Net can still outperform more complex models by adjusting and calibrating the inter-configurations like optimizers and loss functions. Our proposed model was applied to both BraTS 2020 and BraTS 2021 challenge datasets to provide a more generalized analysis. According to BraTS 2020 dataset, the suggested Bridged U-Net achieved average Dice Similarity Coefficients of 0.78, 0.8159, and 0.9073, as well as HD95% values of 21.684, 15.941, and 5.37 for the ET, TC, and WT, respectively. For the BraTS 2021 dataset, it obtained an average DSC of 0.8434, 0.8594, and 0.9187 as well as an average HD95% of 11.669, 14,1887, and 5.3687 for the ET, TC, and WT, respectively. It was found that our model performed well regarding the Enhancing Tumor (TC) sub-region, which is the most complex sub-region in brain tumors and commonly scores a lower DSC value.

- Future directions: The future scope of this work can be accomplished by extending nnU-Net (the winner of BraTS 2020 challenge) to our model for automatizing the process of finding the optimal deep learning model for brain tumor segmentation task and to overcome the tedious hyperparameter tuning process and by trying different dilations of convolutions to adjust the number of parameters. Additionally, the use of transfer learning techniques by using pre-trained models could be explored to further reduce the training time and complexity.

## Figures and Tables

**Table 1:** BraTS 2020, 2021 dataset details.

Dataset	Input Image Size	No. of Training Dataset	No. of Validation Dataset
BraTS 2020	240 × 240 × 155	369	125
BraTS 2021	240 × 240 × 155	1251	219

**Table 2:** The used configurations for all models.

Activation Function	Leaky ReLU
Batch size	2
Epochs	150
Loss function	Dice loss
Optimizer	Ranger-21
Normalization layer	Instance (except the proposed model)
Patch sliding window	[128 128 128]

**Table 3:** Used Experimental Hyperparameters for all models, Bold refers to the best choices.

Hyperparameters	Tested Options				
Optimizer	Adam	Ranger 20	** Ranger 21 **		
Loss function	** Dice **	Dice-CE	Focal	Dice-Focal	Dice-Boundary
Batch size	1	** 2 **	4		
Epochs	100	120	** 150 **	200	300
Normalization layer	Group	** Instance **	Batch	** Evolving Norm **	
Activation function	ReLU	** LeakyRelu **	Mish	PreLU	
Learning rate	0.0002	** 0.0003 **	0.0004		
Patch sliding window	[128 128 128]	[64 64 64]	[32 32 32]	[16 16 16]	

**Table 4:** Ablation study of the optimizer choice impact.

Optimizer	Results (DSC)		
	ET	TC	WT
Adam	0.817	0.853	0.901
Ranger 20	0.825	0.863	0.912
Ranger 21	0.843	0.859	0.918

**Table 5:** Ablation study of the loss function choice impact.

Loss Function	Results (DSC)		
	ET	TC	WT
**Dice**	0.843	0.859	0.918
Dice-CE	0.830	0.858	0.909
Focal	0.825	0.849	0.9067
Dice-Focal	0.8291	0.848	0.9103
Dice-boundary	0.83	0.847	0.893

**Table 6:** Our proposed model variant-2 (5-fold cross validation) results on BraTS 2021 validation dataset.

Method	DSC				Hausdorff Distance 95%				Params	Training Time
	ET	TC	WT	Mean	ET	TC	WT	Mean		
3D_U-Net	0.821	0.868	0.910	0.866	19.311	7.749	4.420	10.493	23 M	23 h
Attention U-Net	0.838	0.861	0.917	0.872	16.028	12.743	4.296	11.022	23 M	23 h
R2_Attention_U-Net	0.836	0.848	0.919	0.867	17.609	14.807	4.128	12.181	24.2 M	23.5 h
Bridged U-Net ASPP_EVO v1	0.843	0.859	0.918	0.873	11.669	14.188	5.368	10.408	22 M	21 h
Bridged U-Net ASPP_EVO v2	0.830	0.858	0.9213	0.870	19.684	10.156	4.449	11.430	22.1 M	21 h

**Table 7:** Performance comparison with 3 state-of-the-art architectures on BraTS 2021 validation dataset (219 cases).

Metric (Mean)	ET	TC	WT
Dice	0.839	0.865	0.925
HD95%	19.167	12.646	3.755
Sensitivity	0.829	0.844	0.931
Specificity	0.999	0.999	0.999
Precision	0.907	0.943	0.927

**Table 8:** Comparative analysis of the state-of-the-art models and the proposed model.

Method	Architecture Features	Limitations
3D_U-Net	Basic 3D U-Net	Limited generalizability
Attention U-Net	Including attention mechanism at the decoder side	High computational complexity
R2_Attention_U-Net	Includes residual connections and recurrent connections	High complexity and time consuming while training and inefficient in class imbalance issue
Bridged U-Net ASPP_EVO v1 & Bridged U-Net ASPP_EVO v2	Includes EVO-Norm layers, ASPP block, bridge connections, and deep supervision	Computational complexity and extra parameters tuning is needed

**Table 9:** Performance comparison of our proposed model with other recent state-of-the-art models.

Author	Dataset	Methods and Remarks	Segmentation Results (DSC)		
			ET	WT	TC
Ellis et al. [43]	BraTS2020	Several Modified U-net architectures (weighting region contours, permuting data, labels grouping) and finally an ensembled model was provided	0.7412	0.8988	0.808
Cirillo et al. [10]	BraTS2020	Vox-to-vox architecture is based on GAN. Generator is a U-Net architecture while the discriminator is a CNN-based model	0.7504	0.8926	0.7919
Isensee et al. [44]	BraTS2020	nnU-Net architecture with augmentation and modification **Winner of MICCAI BraTS 2020 challenge**	0.8203	0.8895	0.850
**Our work**	BraTS2020	**Bridged U-Net_ASPP_EVO (V2)**	**0.78**	**0.907**	**0.8159**
Wang et al. [45]	BraTS2020	Trans U-Net (TransBTS)	0.7873	0.9009	0.8173
Jia et al. [39]	BraTS2020	Hybrid High-resolution and Non-local Feature Network (H2NF-Net)	0.7875	0.912	0.8546
Rahman et al. [46]	BraTS 2021	3D UNet-Context Encoding (UNCE)	0.7787	0.9159	0.8499
Pei et al. [47]	BraTS 2021	3D ResU-Net	0.8190	0.91959	0.8503
Peiris et al. [11]	BraTS 2021	Three modules were used, U-Net, Critic adversarial network, and Virtual Adversarial Training	0.8138	0.9076	0.8538
Jia et al. [48]	BraTS 2021	BiTr-Unet	0.8187	0.9097	0.8434
Luu et al. [49]	BraTS 2021	Extended nn-Unet with a larger U-Net architecture **Winner of RSNA MICCAI BraTS 2021 challenge**	0.8451	0.9275	0.8781
**Our work**	BraTS 2021	Bridged U-Net_ASPP_EVO v1	**0.8434**	0.9187	0.8594
	BraTS 2021	Bridged U-Net_ASPP_EVO v2	0.8395	**0.9251**	**0.8658**

## Data Availability

The dataset used in this work for the experimental results and to support the findings is publicly available from the MICCAI BraTS 2020 challenge, which can be acquired from https://ipp.cbica.upenn.edu/. Access date on 15 December 2022.
